# Supplementary material for: Resolving the full spectrum of human genome variation using Linked-Reads
Source: Genome Res. 2019 Apr;29(4):635–45. doi: 10.1101/gr.234443.118 (PMC6442396; doi:10.1101/gr.234443.118)
Supplement: Supplemental Material [file supp_gr.234443.118_Supplemental_longranger-2.2.zip › longranger-2.2.tar.bz2!/lr2.2-public/lib/stan/cmdstan/stan/doc/api/html/program__grammar__inst_8cpp_source.html]

Stan: src/stan/gm/grammars/program\_grammar\_inst.cpp Source File


|  |  |
| --- | --- |
|  | Stan  2.5.0  probability, sampling & optimization |


- Main Page
- Related Pages
- Namespaces
- Classes
- Files

- File List
- File Members

All Classes Namespaces Files Functions Variables Typedefs Enumerations Enumerator Friends Macros Pages

- src
- stan
- gm
- grammars

program\_grammar\_inst.cpp

Go to the documentation of this file.

1 #include <stan/gm/grammars/program\_grammar\_def.hpp>

2 #include <stan/gm/grammars/iterator\_typedefs.hpp>

3

4 namespace stan {

5  namespace gm {

6  template struct program\_grammar<pos\_iterator\_t>;

7  }

8 }

iterator\_typedefs.hpp

program\_grammar\_def.hpp


---

[ Stan Home Page ]

*© 2011–2014,
Stan Development Team.*
